# Rising to the challenge of surging seas

**DOI:** 10.1038/ncomms16127

**Published:** 2017-07-10

## Abstract

Amid increasingly extreme projections for future sea-level rise, concerns are mounting that policymakers are struggling to keep abreast of fast-paced scientific developments. To ease this burden and increase the accessibility of published research, we have compiled an editor-curated collection of the most recent sea-level rise articles published at *Nature Communications*.

Waking up to the sound of crashing waves, ocean views and sandy beaches remains, for many, the dream. However, according to a series of recent reports and studies on rising sea levels, sleepless nights could be in store for beachfront real-estate owners and residents of coastal cities the world over.

Both researchers and decision makers need to manage the high volume of scientific output currently emerging; a time-consuming task, particularly for sea-level rise research, a field that is constantly evolving and requires timely action

Earlier this year, the United States’ National Oceanic and Atmospheric Administration (NOAA) released a technical report on sea-level rise that included a new extreme scenario. The revised upper estimate warns that US coastlines, currently populated by 12 million residents, could experience a sea-level increase of 2.5 m by the year 2100 if no mitigating action is taken^1^. The extreme projection in part reflects new research on the stability of the Antarctic ice sheet, which, while uncertain, indicates that the southern ice cap could alone contribute as much as 1 m to global sea levels by the end of the century^2^. While this scenario remains highly unlikely, it is not the only study to markedly increase end-of-the-century sea-level rise estimates^3^,^4^. These revisions significantly increase projections provided by the last Intergovernmental Panel on Climate Change (IPCC) assessment report, in some cases more than doubling the upper bound^5^. With the next IPCC report not due until 2021, policy makers are commissioning their own reports in an effort to keep abreast of rapid scientific developments and the associated uncertainties^6^. The timing of decisive action could be crucial in ensuring that coastal defences are adequate. However, it remains difficult, and expensive, to prepare for the worst when future projections continue to change.

Furthermore, the effects of rising sea levels are more than physical in nature and are likely to propagate far from the coast. Rapid economic growth, urban development and migration are expected to increase the number of people living in the low-elevation coastal zone to 1.4 billion by the year 2060 (ref. 7). With sea levels rising three times faster than they were 25 years ago^8^ and recent reports suggesting that an increase as small as 0.2 m could double the risk of coastal storm-related flooding^9^, the potential impact is likely to become increasingly severe. Under an extreme sea-level rise scenario, one study forecasts that as many as 13.1 million US citizens could become climate change refugees, triggering a wave of mass migration away from the coastal zone, resulting in a heavy socioeconomic burden on the infrastructure of inland cities^10^. The cost of sea-level defence is, however, high. A 2014 assessment estimated that annual investments in the range of $12-71 billion would be required to protect against the threat^11^, while inaction could total $1 trillion per year by 2050 for 136 of the world’s largest coastal cities^12^.

With population growth roughly twice the US average and a ribbon of expensive real-estate occupying its low-lying coast, Florida—the state for which NOAA’s new extreme scenario is most worrying—is well aware of the multifaceted nature of the sea-level rise problem. Submerged highways, flooded basements and backed-up sewage systems are an increasingly common occurrence for residents of the sunshine state. Two years ago, in an effort to find solutions to the problem, Florida International University launched their Sea Level Solutions Centre (http://slsc.fiu.edu/). The centre, which combines expertise in the natural, physical and social sciences, is tasked with focusing on the science behind sea-level rise and is working with all stakeholders from scientists and the public to the government and the private sector in order to research, develop, plan and finance long-term sustainable strategies to combat rising seas.

Such interdisciplinary initiatives are, however, not yet the norm, and the science behind sea-level rise can often be disparate and difficult to access. At *Nature Communications*, we are committed to supporting research on the grand challenges facing society, and we encourage studies that not only investigate the physical nature of sea-level rise, but also evaluate the socio-economic effects and propose sustainable solutions. Although all articles published in *Nature Communications* are fully accessible upon publication, we feel more is needed to enhance the visibility of relevant research to invested parties. Both researchers and decision makers need to manage the high volume of scientific output currently emerging; a time-consuming task, particularly for sea-level rise research, a field that is constantly evolving and requires timely action. In support, this month we launched our sea-level rise collection [ http://www.nature.com/collections/ncomms-sealevelrise]—a periodically updated dedicated space to sea-level rise related research published by *Nature Communications*. We hope the collection will provide a useful resource for all stakeholders involved in assessing and mitigating sea-level rise, from scientists working on the next IPCC report to local policymakers in need of up-to-date science to inform their decisions.

Sea-level rise is still perceived by many as a distant problem, and, given the associated expense and scientific uncertainty, caution in implementing mitigating solutions is understandable. However, this is not the case for all. While recent events, such as hurricane Sandy, have acted as a warning of the increasing threat to the US and have instigated preparatory action, such action did not come soon enough for the evacuated residents of the Taro atoll of the Pacific Ocean’s Solomon Islands (https://www.scientificamerican.com/article/township-in-solomon-islands-is-1st-in-pacific-to-relocate-due-to-climate-change/). In light of the increasing pressures from rising seas the low-lying small island nations campaigned, successfully, for limiting warming to 1.5 °C above pre-industrial levels in the now ratified Paris Agreement. It is by reducing and limiting emissions that we will ultimately avoid extreme sea-level scenarios and achieve the first step to protecting vulnerable coasts, whether it be luxury beachfront resorts, such as President Trump’s Mar-a-Lago estate in Florida, or the idyllic atoll homes of the Pacific Island nations. We should prioritize efforts to avoid dangerous climate change, while at the same time continuing to inform all stakeholders of the likelihood of extreme sea-level rise—being prepared is a decision we are unlikely to regret.

Published online: 10 July 2017
